# Transfusion of Blood and Infusion of Saline Solutions: Indications, Methods and Results

**Published:** 1894-11

**Authors:** Valentine H. Taliaferro

**Affiliations:** Atlanta, Ga.; 186 S. Pryor Street


### TRANSFUSION OF BLOOD AND INFUSION OF SALINE SOLUTIONS: INDICATIONS, METHODS AND RESULTS.*

By VALENTINE H. TALIAFERRO, M. D., ATLANTA, GA.

My chief object in presenting this paper is for the purpose of calling to your attention and soliciting your interest in a means of saving life, which in certain instances proves the most powerful, the quickest and often the only means capable

*Read before the Tri-State Medical Association.

of accomplishing any good; and to briefly review the subject for the benefit of those who are unfamiliar with the methods now employed.

Magnus Pegil, in 1604, published a work in which he describes the operation of transfusion, being sixteen years previous to the discovery of the circulation by Harvey.

Deneys, of Paris, performed the first transfusion on the human subject of which we have any knowledge, using nine or ten ounces of lamb's blood, which he injected into the basilic vein "for repeated bleedings, with most gratifying results." Deneys afterward operated upon Antoine Maurey, who was suffering from periodic mania. The first two operations greatly improved the man, but while transfusing him the third time he expired. Deneys was tried for manslaughter, but not convicted. The operation was condemned by the court and dropped into disuse for nearly one hundred years.

Many unsuccessful attempts to revive the operation were made, but it was not until early in the present century that Dr. Blundell, of England, revived the operation, using human blood in preference. Such success met his efforts, and the efforts of those who followed him, that it became a recognized therapeutic method the world over and has remained so to the present day.

The blood of various animals, human blood, cow's and goat's milk, plain water, wine and saline solutions have all been used as intra venous injections. But from the past experience of our best experimenters, we are now able to condemn all but human blood and the normal saline solution.

When the blood of the inferior animal is introduced into the veins of man, it undergoes rapid dissolution. The hæmaglobine is first set free and changes the circulating fluid to a ruby red color. The corpuscles are entirely dissolved. Potass, creatin, leucin and other substances capable of doing great harm are also liberated.

Very distressing symptoms and often fatal results follow the use of lamb's or calf's blood when over four or five ounces are used, and this small quantity is capable of doing no good.

Vomiting, chills, convulsions, hæmaturia, albuminuria and uræmic poisoning which often occur, are easily explained by the dissolution of the corpuscles and the irritating effect of the free hæmaglobine.

Plain water, even when in small quantities, quickly exerts a very deleterious action upon the whole of the circulating fluid and will rapidly produce death. The blood plasma is diluted and soon loses its normal alkalinity. By its physical action on the corpuscles, they become swollen and pale, the hæmaglobine is dissolved out and their vitality lost. Wine and milk are little less dangerous.

In transfusing blood, only human blood is to be used. Every tissue in the body being dependent for its nutriment upon the blood, it is apparent to all that to supply nutrition to the various organs, especially to the heart and brain, we must first improve the character of the blood. This is often in extreme cases impossible by the ordinary therapeutic measures. By introducing directly into the circulation a sufficient quantity of healthy blood, we at once accomplish the end in view. To the heart, there is no more powerful stimulant than the red corpuscle. From ten to fifteen ounces should be used at a time. Many diseases have been treated with tranfusions of blood, some with no success, some with doubtful success, but many with decided benefit. It is the latter in which we are interested and I briefly mention a few. Most amenable to this remedy are those diseases which are directly dependent upon a diseased condition of the blood itself.

In simple anæmia and chlorosis which are persistent and fail to yield to the ordinary therapeutic measures, the repeated transfusions of small quantities of blood give most gratifying results. In one case of this kind which I watched with the greatest interest, one transfusion of about ten ounces of blood benefited the patient to such a degree that it was deemed unnecessary to repeat the operation.

Pernicious anæmia, leucocythemia and hæmophilia have all been treated in this way with sufficient success to warrant the operation in extreme cases. In severe carbonic oxide poisoning, transfusion often offers the only chance of recovery. The gas, both by its poisonous effect upon the blood into which it is rapidly absorbed, and its action on the lungs, preventing oxygenation and decarbonization, causes to be retained in the blood this double amount of gas. To avoid over-repletion and to remove as much of the poison as possible, blood should be first abstracted and the same amount of pure blood then introduced. This should be repeated several times, or until a sufficient amount of poison has been eliminated to assure the patient's recovery. Many cases of impending death have been thus averted.

In persistent vomiting, over-lactation, excessive diarrhœa and other diseases which have produced irreparable exhaustion, transfusion, if not deferred too long, offers every hope of success.

Several cases of asphixia neonatorum have been successfully treated by using the blood from the placenta. In profuse hemorrhage from the bowels and collapse of typhoid fever, nothing else offers the same chance of success as blood, and many such cases are reported in which it has been the means of arresting the approach of death in these much dreaded compliactions of typhoid.

Equally efficacious in other conditions is the effect of the normal salt solution. Being less dangerous in its use and more easily procured than blood, it has largely taken its place in a a number of instances in which blood was formerly used.

A usual fatal complication or termination of diabetes melitus is diabetic coma. More than one-half the cases of diabetes terminate fatally in this condition. The coma is generally brought about suddenly by excessive exertion, great mental strain or nerve depression. The two theories most generally accepted upon which this symptom is immediately dependent, are the want of alkalinity of the blood and the absorption from the alimentary canal of toxic substances. The presence of acetone in the blood is perhaps another cause. But setting theory aside, certain it is that in the coma of diabetics there is great decrease in the alkalinity of the blood and that by correcting this abnormal condition, we are favoring to the best of our ability a prolongation of life. By restoring to consciousness and staying the advent of death even for a short while, sufficient time may possibly be gained to re-establish renal activity and thereby avoid a fatal termination. Here, it is the saline infusion upon which we must depend. In profound diabetic coma, we can hope for but little permanent good from this measure, but even here we can bring our patients into a state of consciousness lasting from a few minutes to hours and occasionally with permanent benefit. It is in coma of milder degree and especially in threatened coma that the saline acts most satisfactorily, but it must be used early and in large and repeated quantities.

In the recent epidemics of cholera, in those cases of rapid waste and collapse from exhaustive diarrhœa, saline infusions have been extensively used and with great benefit.

But it is chiefly in the treatment of shock and profuse hemorrhages that both blood and the salines have their greatest field of usefulness, acting here with truly magical effect. Success so surely follows this method in hemorrhages from any cause which are so profuse as to immediately threaten life, that we are guilty of gross neglect who fails to offer this chance of recovery to any who are *in extremis.*

After one has bled to death, over one-half of the blood still remains in the vessels, which is sufficient to sustain life, if it can be kept in circulation.

When blood is lost the vaso motor nerves cause the vessels to contract in proportion to the amount lost, keeping up the normal pressure. Beyond a certain point this compensation fails and the pressure falls with great suddenness. By increasing the bulk of circulating fluid sufficiently to produce the proper tension in the vessels, the heart will resume and continue its work. For this purpose, the salt solution is preferable. It can be used with more safety, procured more speedily, used in larger quantities and at a temperature which powerfully stimulates the heart and contracts the arteries.

It is the obstetrician and gynecologist who are so often called upon to combat those sudden, profuse and rapidly fatal hemorrhages to whom this life-saving means should most strongly appeal. How many poor women have bled to death! I dare say there is not one amongst us who has not seen such an ending. Standing in the presence of this grim and deadly enemy, who has not felt powerless to loose its dreaded grasp. With the means now at our command, we can fill and contract the emptied vessels, stimulate the heart, keep up the circulation, stop the bleeding and restore to safety those who are in imminent peril.

Equally as efficacious as in the treatment of hemorrhage is the saline infusion in the treatment of shock, acting practically in the same way.

In prolonged surgical operations it is not the hemorrhage which we dread, for this can usually be controlled, but it is the inevitable shock which is so often rapidly fatal.

There is nothing known to us which is so potent in its relief, so rapid in its action, or so certain in its effect, as large infusions of hot salt solution.

The best method of performing transfusion or infusion is of no little importance. There are very many instruments and methods now in use for which superior advantage is claimed. The simplest and safest is the one which deserves our attention, the one to which we should give preference. The direct or immediate method of transfusing blood with an Aveling's apparatus is much the best. In this way, blood from the donor flows directly to the recipient. Dr. Aveling invented his simple instrument in 1864, and with little modification it has remained the best to the present day. It consists of a rubber syringe made in one piece, similar to a Davidson, but with smaller bulb, and without valves or joints upon which the blood can clot; each end is provided with a silver canula and stop-cock. By knowing the capacity of the bulb, the amount of blood transfused can be easily determined. A prominent vein at the bend of the arm, preferably the median basilic, is usually chosen, though I prefer to go below the elbow, thus allowing the patient to bend the arm at will after the operation, without pain or danger to the incision.

Through an incision half an inch in length the vein can be sufficiently exposed. Great care is necessary not to lose any of the patient's blood. To prevent this a ligature is passed around the distal end of the vein and tied in a single knot. But one ligature is necessary, the valves preventing the backward flow of the blood. A longitudinal incision is made with a sharp-pointed bistoury into the vein, in proportion to the size of the canula used. I prefer this to the V shape incision, as there is less danger of entirely severing the collapsed vein, and the incision is more easily united.

The syringe being completely filled with a weak salt solution, the stop-cock turned to prevent its escape, the receiving canula is introduced into the vein of the donor pointing to the hand, the stop-cock in the delivering end is then turned to allow the fluid in the syringe to flow, the bulb is slowly pressed, as the canula is introduced into the recipient's arm in direction of the shoulder. This precaution is to prevent the possible entrance of air into the vein. By the alternate action of the stop-cocks, which take the place of valves, the syringe is repeatedly filled and emptied, until a sufficient quantity is introduced. When the canula is removed, a continuous suture closes the skin incision. Before the stitches are tightened, the ligature, which is tied in only one knot, is easily slipped off.

For direct transfusion, another instrument is that of Roussel; but, this is entirely too complicated for general use, nor does the syringe of Grailey Hewitt meet the requirements. There are several ingenious methods of using defibrinated blood, but when blood is to be used, and can be had, the direct method is always to be chosen.

Aveling's apparatus is also the best when the salt solution is used. With this instrument the speed with which the fluid is introduced can be more accurately regulated, the quantity more easily measured and the tension of the circulation more readily determined. All of these are important points. To avoid over-distention of the heart and too great dilution of the blood, the fluid should be introduced slowly, not exceeding one pint in five minutes. Four or five pints are required in the worst cases, rarely less than two. A few ounces are of no avail, but the quantity must be determined at the time of the operation by the effect produced. By the resistance to the fluid infused, the tension of the circulation is communicated to the hand which presses the bulb, enabling us in this way to avoid over-distention. The pulse also affords valuable information, and should be carefully observed during the operation.

When we remember that with a saline solution the mass of blood can be increased to six times its normal amount without producing death from plethora, it should encourage us to use without fear a sufficient quantity of fluid to at least fill the depleted vessels.

Here again there are several methods of using the saline solution. Dr. Howard Kelley uses an inverted aspirating bottle with blunt needle and by reversing the current of air through the bottle forces the fluid out. Dr. Dawbarn, of New York, uses an ordinary hypodermic needle attached to a Davidson syringe, inserting the needle without any incision directly into the common femoral artery. Another instrument frequently used consists of a funnel, tube and canula. But the method with Aveling syringe, which I have described at length, has many advantages which none of the others possess.

It is important to preserve the normal alkalinity of the blood and for this purpose different salts are used. Plain water should never be used. One drachm of sodium chloride to the pint of sterilized water, which being strained and heated to 110 or 115 F., is all that is necessary. As pointed out by Dr. Dawbarn, the heat is an important factor in the stimulating qualities of the fluid and should never be omitted.

The dangers and difficulties of this operation have been greatly over-estimated, and it is perhaps on this account that it is so rarely resorted to in this section of country. Even though it were dangerous, we should not be deterred from using it in those extreme cases where heroic measures are always justifiable.

I have made this operation five times, have assisted in several others and have not yet seen an evil result follow. Nor have I seen a single case where the remedy has failed to accomplish some good. Like other remedies which are considered difficult in their administration or dangerous in their use, it is often delayed until too late to hope for success. If used in time, before the vital functions are damaged beyond repair, we can, in the great majority of cases, be assured of success. I could detail without end cases of shock and hemorrhage reported by our ablest surgeons which have been saved from death by the prompt transfusion of blood or saline solution. Those who have tried it are unanimous in giving their hearty support. I will only report two cases, one to illustrate the effect of the salt solution in shock, the other in hemorrhage.

The first case was one of great shock following vaginal hystereotomy for carcinoma. The woman was extremely weak when the operation was begun and came very near dying while on the table. The operation lasted for over two hours. She was put to bed cold, clammy and pulseless, the physicians present expecting her to die at any moment--all the usual stimulants were tried in vain; she failed to rally. About three hours after the operation, I infused into her veins two pints of warm salt solution. During the infusion the pulse became perceptible in the opposite arm; the patient gained consciousness, and slowly but steadily improved. Never before have I seen a case recover from such profound collapse.

The second case was one of hemorrhage from an attempted suicide with a razor. This man was brought into St. Bartholomew's Hospital, completely exsanguinated and pulseless. His condition was deemed hopeless by all present, but the house surgeon, whom I assisted, decided to give him the chance of an infusion. He revived while the operation was in progress and soon regained his health.

It is not because I offer anything new that I make these remarks, but with the hope that I may impress you with the value of this all important remedy and to invite your free discussion.

*186 S. Pryor Street.*

